# Inappropriate study inclusion in meta-analysis of sham-controlled rTMS for treatment-resistant depression

**DOI:** 10.1186/s12888-024-05703-5

**Published:** 2024-04-02

**Authors:** Kevin P. Kennedy

**Affiliations:** Department of Psychiatry, UCLA Semel Institute for Neuroscience and Human Behavior, University of California Los Angeles, Los Angeles, USA

**Keywords:** Sham-control, rTMS, Treatment-resistant depression

## Abstract

Dr. Vida and colleagues have published an important meta-analysis on a critical topic in psychiatry: the efficacy of double-blind, sham-controlled rTMS in treatment-resistant depression (TRD) [1]. The primary reported finding was a significant effect of rTMS on remission and response (RR 2.25 and 2.78 respectively) compared to sham rTMS. A close evaluation of the studies included in this meta-analysis raises concerns about the accuracy of these findings.

Dr. Vida and colleagues have published an important meta-analysis on a critical topic in psychiatry: the efficacy of double-blind, sham-controlled rTMS in treatment-resistant depression (TRD) [1]. The primary reported finding was a significant effect of rTMS on remission and response (RR 2.25 and 2.78 respectively) compared to sham rTMS. A close evaluation of the studies included in this meta-analysis raises concerns about the accuracy of these findings.

In both the abstract and methods sections, the authors specify that only randomized, sham-controlled trials of patients with at least two antidepressant treatment failures were included. The abstract specifies that included studies were double-blind, but this is not explicitly listed as inclusion criteria in the methods section.

Of the 19 studies included in the random-effects meta-analysis, the study by Filipčić et al. was weighed as 43.48% for response and 33.56% for remission, by far the largest and most impactful study in each analysis [2]. This study reported significant positive findings for rTMS for response (RR 2.35) and remission (RR 4.59). However, it did not meet the stated inclusion criteria and should not have been included in the meta-analysis.

This study was a single-blind comparison between two active FDA-approved rTMS modalities with a control group that received standard pharmacotherapy *without* sham TMS. To quote directly from the study: “228 MDD patients were randomized to 20 sessions of H1-coil or 8-coil as an adjunct to standard-of-care pharmacotherapy, or standard-of-care pharmacotherapy alone” and “we did not use a sham-control TMS coil.” Aside from the lack of double-blinding and sham-control, subjects in the rTMS arms were monitored daily while the pharmacotherapy arm only received an evaluation at baseline and at 4-weeks.

There is evidence that TMS can induce a large placebo response that can only be controlled for by a high-quality sham and appropriate blinding [3, 4]. The inclusion of this heavily-weighted study risks inflating the overall estimate of efficacy for rTMS in TRD, given that its highly positive findings may have been caused by the single-blind design and absence of a sham-control (much less other fundamental differences, like the frequency of visits).

Given the importance of this subject, this meta-analysis would benefit from a re-analysis that excluded the Filipčić et al. study to reflect the outcomes only of double-blind, sham-controlled trials, in accordance with the meta-analysis’s stated inclusion criteria. At a minimum, this study should be removed from the meta-analysis so that researchers and clinicians have an accurate understanding of the number of patients included in the highest-quality studies of rTMS in TRD—those that are truly double-blinded and sham-controlled. Readers should be aware that most rTMS trials for TRD have been quite small–only two had more than 45 subjects in this meta-analysis, excluding Filipčić–and many have had unclear (rather than low) risk of bias [5].

## Data Availability

Not relevant.

## Declarations

### Authors’ information

Not relevant.
